# 18^th^ Congress of the Academy for Multidisciplinary Neurotraumatology: 26-28 February 2020 | Cairo | Egypt

**DOI:** 10.25122/jml-2020-1004

**Published:** 2020

**Authors:** Dragos Cretoiu

**Affiliations:** Carol Davila University of Medicine and Pharmacy, Bucharest, Romania

## Introduction

**Figure 1:**
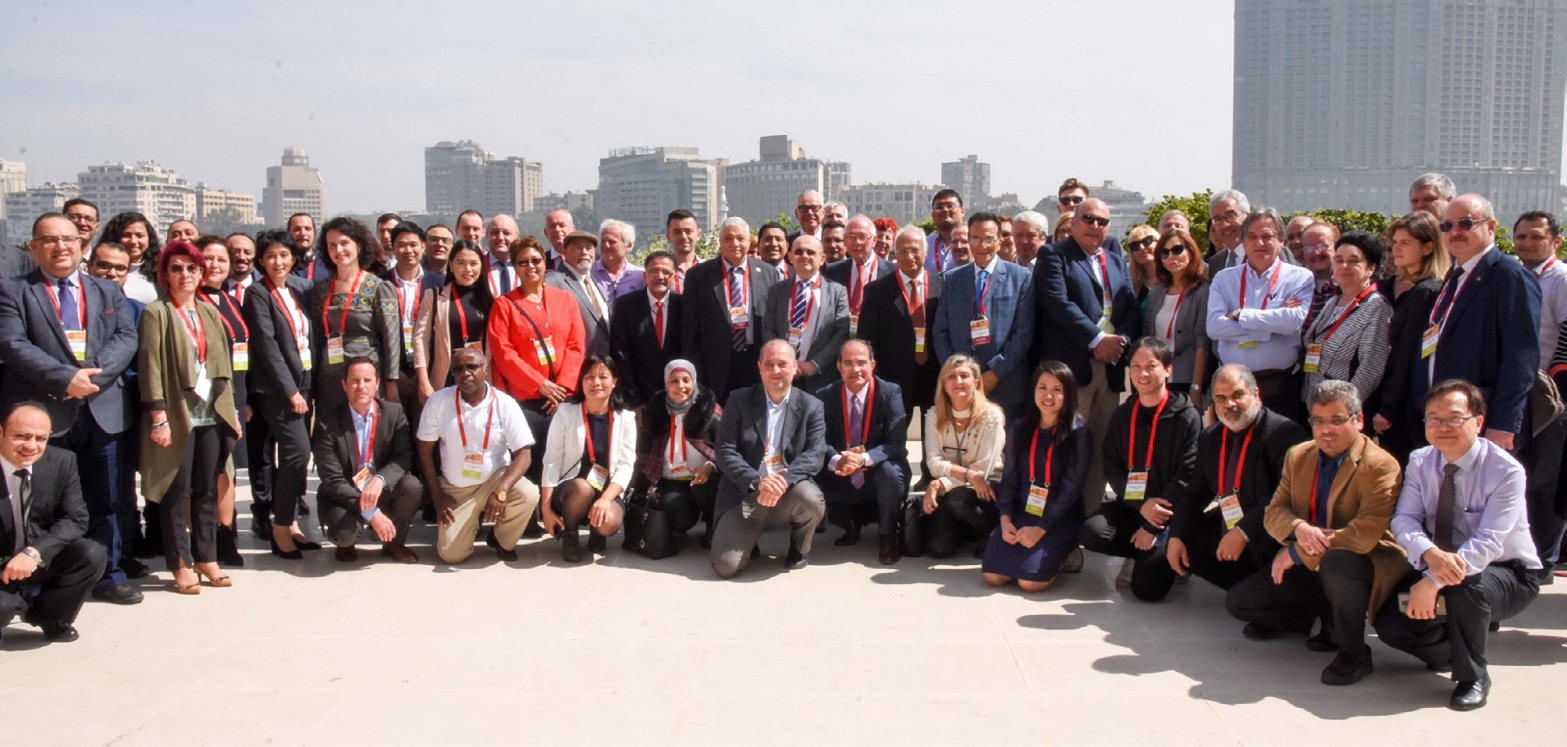
Group photo of the 18th Congress of the Academy for Multidisciplinary Neurotraumatology

The 2020 Congress of the Academy for Multidisciplinary Neurotraumatology (AMN) took place between February 26-28 in Cairo, Egypt. This educational event brought together 150 participants from 19 countries, specialized in multiple fields, including neurosurgery, neurology, neurorehabilitation, psychology, and public health (Figure 1). The event set the stage for two days of heated debate on a broad range of issues and challenges in clinical neurotraumatology and neuroscience, promoting the integration of keynote lectures presenting novel scientific evidence, with teaching-oriented workshops and round table discussions. A rich and diverse audience of healthcare professionals interested in this steadily expanding and multidisciplinary field attended the event: physicians, nurses, therapists, public health experts, and clinical researchers. Exciting lectures on various topics were covered in four presentation sessions, three-country sessions, and a half-day group workshop.

## Congress Sessions

In the opening, the presidential session was chaired by two local representatives: Dr. Mohamed S. El-Tamawy (Cairo University, Cairo, Egypt), and Dr. Anwar El Etribi (Ain Shams University). Dr. Dafin F. Mureșanu from Romania, President of the European Federation of Neurorehabilitation Societies (EFNR), presented the results of the CAPTAIN trial series and meta-analysis. CAPTAIN I and CAPTAIN II trials explored the efficacy of the neurotrophic factors for recovery after traumatic brain injury (TBI). The results of the two trials indicate significant results in moderate-severe TBI recovery and confirm an excellent safety profile for the intervention.

The second lecture introduced the audience to a new methodology for TBI clinical research - the multidimensional approach based on the Wei-Lachin pooling procedure. AMN President Dr. Johannes Vester from Germany made the case that multidimensional analysis opens an entirely new direction for clinical and statistical thinking, and is perhaps much closer to the intricate outcomes after traumatic brain injury than the previous “one-criterion paradigm”, which ruled clinical research on neuroprotective treatments for the last decades. Further on, Dr. Mohamed El-Tamawy (Cairo University, Egypt) guided the audience on a visit thorough neurology in Ancient Egypt. This history lesson provided a better understanding of the beginnings of this medical branch and the early studies on the nervous system. The last lecture of the session focused on cognitive rehabilitation after TBI, and it was held by Dr. Volker Homberg (Germany), Chairman of AMN Scientific Program Committee, and also Secretary-General of the WFNR and EFNR.

Session 1 began with a topic dedicated to adjunct management of chronic subdural hematoma, presented by Dr. Wai Sang Poon (Division of Neurosurgery, Prince of Wales Hospital, The Chinese University of Hong Kong, Hong Kong), as a review of the literature on suggested treatments which reduce surgical recurrence. He was followed by Dr. Peter Lackner from Austria, who focused on the possibilities and limitations of registry data analyses. Various approaches for identifying optimal outcome parameters sets were discussed. The third lecture was delivered by the Egyptian Dr. Essam Emara, presenting his decade-long experience in using neurotrophic factors in neurosurgical disorders as a protective agent against surgical trauma.

Session 2 was opened by Dr. Christian Matula (Austria), who spoke about the changes that have occurred in neurotraumatology in the last years. Modern neurotraumatology nowadays has proven to be the sophisticated approach of multidisciplinary teams consisting of experts from different disciplines, with the explicit mission to advance health worldwide in brain-injured patients significantly. The overall goal of this endeavor is to provide the highest level of neurotraumatology care for patients, translate fundamental neuroscience into clinical practice, and train the next generation of clinical scientists. The second speaker of the session, Dr. Ignacio Previgliano (Argentina), provided insights from acute brain trauma management in the intensive care unit (ICU), pointing out disparities in patient outcomes due to various levels of national or regional health systems. He also presented the role of Point-of-Care Ultrasound in TBI management as a strategy for ICU care in low-resource settings. Next, Dr. Pieter Vos from the Netherlands brought into the spotlight the importance of clinical decision rules and biomarkers needed to accurately detect the intracranial injury and identify patients with an increased risk for intracranial hematoma. Finally, Dr. Felix Brehar (Romania) presented a retrospective multi-centric cohort study on 7769 adult patients with TBI, which evaluated the effects of neurotrophic factors on the outcome after TBI.

Session 3 began with a keynote from Dr. Hassam Hosny from Egypt, who approached clinical management issues in post-traumatic seizures and epilepsy. The presentation of this session was held by Dr. Dana Boering from Germany, who addressed the neuromodulation strategies used in TBI early and post-acute rehabilitation, such as pharmacological neuromodulation, non-invasive brain stimulation, brain-computer interfaces, real-time fMRI neurofeedback, as well as a short glance at current behavioral neuromodulation techniques (i.e., somatosensory input reduction/augmentation, combined cortical/peripheral stimulation, virtual reality techniques, robotics), outlining benefits as well as caveats and future development fields.

In the end, Stefan Strilciuc (Romania) presented new concepts in the economic evaluation of pharmacological intervention after traumatic brain injury. Due to the increasing scarcity of health resources, the critical question of cost-effectiveness arises in the context of novel TBI therapeutic approaches. The lecture explored the feasibility and implications of using combined outcome measures to produce an aggregated cost-effectiveness indicator for TBI interventions.

## Country Presentations

To showcase the international perspective of TBI care standards and infrastructure worldwide, the Scientific Committee designed five core domains that cover the most critical issues in this filed:

1. Epidemiology of TBI
2. TBI management protocols
3. TBI care status
4. Improving TBI care – objective and milestones
5. Potential activities to improve TBI care in the near future.

Representatives from all 19 participating countries registered to lecture on these topics, a good indicator for local communities’ initiative in having their issues heard globally, in hopes of improving TBI care in their area of practice. Three individual sessions were accommodated during the first day of congress:

• Session 1: Egypt, Vietnam, Uzbekistan, Ukraine, Russian Federation, Thailand, Jordan
• Session 2: Romania, Poland, Myanmar, Azerbaijan, Georgia, the Philippines
• Session 3: Argentina, Austria, Hong Kong, Kenya, Libya, and Mexico.

## Group Workshops

Group workshops were the essential highlights of the conference, bringing together top-tier specialists to discuss future AMN perspectives. The exercise was organized around six groups, each one focusing on a different topic. The session was chaired by Dr. Johannes Vester (Germany), Dr. Dafin Muresanu (Romania), Dr. Peter Lackner (Austria), and Stefan Strilciuc (Romania).

The first international group of experts drew up a set of proposals on the content the AMN website should offer:

• Dissemination of existing and in-development TBI guidelines and protocols;
• Scientific literature database;
• Statistics on the situation of TBI worldwide;
• Patient-centered sections;
• Virtual teaching courses;
• A discussion forum where the AMN members can discuss and exchange opinions on different medical cases.

The second group offered solutions on how to reduce “door-to-treatment” time, and how the AMN can support the implementation of these suggestions. These include course series for paramedics and communication channels (e.g., a mobile application) liked to a remote specialist who could help with patient assessment and referral to appropriate care settings. 

The third group’s topic revolved around building a multidisciplinary neurotraumatology team hospitals. The group described three existing scenarios:

• Larger, more developed hospitals, where all required disciplines to form the multidisciplinary team exist (very few cases);
• Settings where neurosurgery wards are separate from the hospital where the patient is admitted;
• Settings where patients cannot be adequately treated due to unqualified staff and poor infrastructure.

On team leadership, there was no definite conclusion from the group, as representatives advocated for either neurologist, neurosurgeon, or intensivist. In the spirit of cooperation, the group concluded that the best way to ensure proper treatment and care for the patient is that all disciplines work together and coordinate with no appointed team leader.

The fourth group work mapped care pathways for the patient after TBI, showcasing usual barriers in real-life, practical conditions:

• Scarcity of ambulance services;
• Lack of multidisciplinary teams to assess the patient;
• Poor availability of imaging services;
• Access to neurosurgeons;
• Absence of advanced rehabilitation procedures, such as speech therapy for aphasia patients.

Group 5 focused on the practical aspects of center enrolment in TBI registries. The experts within this group identified several matters to be considered by the AMN:

• legal frameworks, which can be an obstacle in some countries (informed consent, the confidentiality of the data, and ownership of the data);
• who and when should fill the data, how to enroll more centers;
• situations where the patient is transferred from the ambulance to a hospital which is outside the scope of the registry;

The AMN is currently piloting PRESENT (Patient Registry – Short, Essential NeuroTrauma (PRESENT), an academic registry which aims to combine the collection of clinical, injury prevention and quality of care components related to TBI, in the attempt to provide information for the improvement of healthcare delivery, and also for better understanding brain injury in general. With this initiative, the AMN hopes to facilitate an enhanced environment for the dissemination of treatment guidelines, optimal patient pathways, and best practices for neurorehabilitation.

The sixth and final group was assigned the task of formulating recommendations on how the community may develop a strong global impact. Ideas were well received by the AMN Management Committee, with a follow-up pledge on such developments, at future events.

This event was organized by the Foundation of the Society for the Study of Neuroprotection and Neuroplasticity (SSNN) together with its academic partners: The University of Medicine and Pharmacy – Iuliu Hatieganu, Cluj Napoca, Romania; RoNeuro Institute for Neurological Research and Diagnostic, Cluj Napoca, Romania; Romanian Academy of Medical Sciences; Foundation of the Jurnal Medicine and Life, Romania; Amity University, India; Banaras Hindu University, India; The Romanian Society for NeuroRehabilitation. 

The lectures presented during the event highlighted the scientific and clinical knowledge of this complex area and shared the new trends regarding the multidisciplinary approach. The detailed program of the congress and available lectures from the event are available on the AMN website.

